# Characterization of SNPs Associated with Prostate Cancer in Men of Ashkenazic Descent from the Set of GWAS Identified SNPs: Impact of Cancer Family History and Cumulative SNP Risk Prediction

**DOI:** 10.1371/journal.pone.0060083

**Published:** 2013-04-03

**Authors:** Ilir Agalliu, Zhaoming Wang, Tao Wang, Anne Dunn, Hemang Parikh, Timothy Myers, Robert D. Burk, Laufey Amundadottir

**Affiliations:** 1 Department of Epidemiology and Population Health, Albert Einstein College of Medicine, Bronx, New York, United States of America; 2 Cancer Genomics Research Laboratory (CGR), Division of Cancer Epidemiology and Genetics, National Cancer Institute, National Institutes of Health, Bethesda, Maryland, United States of America; 3 Department of Pediatrics (Genetics), Albert Einstein College of Medicine, Bronx, New York, United States of America; 4 Laboratory of Translational Genomics, Division of Cancer Epidemiology and Genetics, National Cancer Institute, National Institutes of Health, Bethesda, Maryland, United States of America; 5 Microbiology and Immunology, Albert Einstein College of Medicine, Bronx, New York, United States of America; 6 Obstetrics, Gynecology and Women’s Health, Albert Einstein College of Medicine, Bronx, New York, United States of America; Baylor College of Medicine, United States of America

## Abstract

**Background:**

Genome-wide association studies (GWAS) have identified multiple SNPs associated with prostate cancer (PrCa). Population isolates may have different sets of risk alleles for PrCa constituting unique population and individual risk profiles.

**Methods:**

To test this hypothesis, associations between 31 GWAS SNPs of PrCa were examined among 979 PrCa cases and 1,251 controls of Ashkenazic descent using logistic regression. We also investigated risks by age at diagnosis, pathological features of PrCa, and family history of cancer. Moreover, we examined associations between cumulative number of risk alleles and PrCa and assessed the utility of risk alleles in PrCa risk prediction by comparing the area under the curve (AUC) for different logistic models.

**Results:**

Of the 31 genotyped SNPs, 8 were associated with PrCa at p≤0.002 (corrected p-value threshold) with odds ratios (ORs) ranging from 1.22 to 1.42 per risk allele. Four SNPs were associated with aggressive PrCa, while three other SNPs showed potential interactions for PrCa by family history of PrCa (rs8102476; 19q13), lung cancer (rs17021918; 4q22), and breast cancer (rs10896449; 11q13). Men in the highest vs. lowest quartile of cumulative number of risk alleles had ORs of 3.70 (95% CI 2.76–4.97); 3.76 (95% CI 2.57–5.50), and 5.20 (95% CI 2.94–9.19) for overall PrCa, aggressive cancer and younger age at diagnosis, respectively. The addition of cumulative risk alleles to the model containing age at diagnosis and family history of PrCa yielded a slightly higher AUC (0.69 vs. 0.64).

**Conclusion:**

These data define a set of risk alleles associated with PrCa in men of Ashkenazic descent and indicate possible genetic differences for PrCa between populations of European and Ashkenazic ancestry. Use of genetic markers might provide an opportunity to identify men at highest risk for younger age of onset PrCa; however, their clinical utility in identifying men at highest risk for aggressive cancer remains limited.

## Introduction

Prostate cancer (PrCa) is the most commonly diagnosed solid tumor among men in developed countries [1], [2]. This cancer has a complex, multi-factorial etiology with an estimated 42% of disease variation being attributed to genetic factors and 58% to environmental/lifestyle factors [3], [4]. One of the strongest risk factors for this disease is family history of PrCa; having a first-degree relative diagnosed with PrCa is associated with a two- to three-fold elevation in the relative risk (RR), and both early age at diagnosis and multiple affected family members are important predictors of risk in relatives [5]–[7]. Taken together, these results suggest an important inherited component to disease risk.

Nevertheless, deciphering the genetic basis for PrCa has been challenging, particularly since unique high-risk genetic mutations have not been identified. The most promising results have emerged from genome-wide association studies (GWAS) of PrCa, which have identified numerous highly replicated and independent SNPs distributed throughout the human genome [8]–[20]. These SNPs individually confer modest risks of PrCa (ORs of 1.05–1.30) and only a subset has been associated with aggressive/metastatic PrCa [21]–[23]. In addition, some risk alleles affect serum prostate specific antigen (PSA) levels [24], [25], which may impact PrCa screening. The set of currently characterized SNPs identified through large GWAS, however, do not explain the majority of the familial/hereditary risk for PrCa [26]–[28].

To date, at least 40 SNPs distributed throughout the genome individually increase the risk of PrCa. Most have been replicated in multiple populations including African-Americans and Asians [29]–[31]. A recent study investigated the associations between a subset of these GWAS SNPs and risk of PrCa in men of Ashkenazic descent [32]. Nine of the 29 SNPs that were investigated in that study were associated with PrCa risk at a nominal p<0.05, and three SNPs remained significant after correction for false-discovery. However, this study did not examine whether risk varied by age at diagnosis, family history of PrCa or pathological features of this disease [32]. Although men of Ashkenazic descent are predominantly of European ancestry, genetically they constitute a unique group with a strong founder effect, and different allele/haplotype frequencies, as well as, distinct linkage disequilibrium profiles [33]–[36], that may affect risk of PrCa. Furthermore, it has been argued that the more homogeneous genetic background of a founder population is advantageous in studying complex diseases (e.g. PrCa) that have a large locus heterogeneity, since confounding by population stratification is reduced [33], [37].

In this report, we present the analyses between 31 SNPs selected from previous GWAS of PrCa using samples from a large case-control study of PrCa in 2,230 men of Ashkenazic descent. In addition, associations were evaluated by age at diagnosis, family history of PrCa and other cancers, and histopathological characteristics of the tumors. We identified a group of risk alleles that are significantly associated with PrCa in this founder population. Furthermore, we demonstrate that several GWAS SNPs are potentially associated with family history of PrCa and other common cancers, which may suggest a complex network of inherited cancer risk syndromes still to be defined. To evaluate the cumulative genetic burden, we also investigated associations between cumulative number of risk alleles and risks of overall PrCa, disease aggressiveness, age at diagnosis and family history of PrCa. We report that men with the most risk alleles (highest quartile) have the highest risks compared to those with the least number of risk alleles (i.e., lowest quartile). Genetic medicine has the potential to identify individuals at risk prior to the decades required for the development of cancer or the manifestation of cancer family history. Nevertheless, further research is needed to better understand how this information could be useful in clinical practice to identify men at highest risk and in reducing cancer morbidity and mortality.

## Materials and Methods

### Study Population

Detailed description of the study population, recruitment methodology and data collection procedures have been described previously [38], [39]. Briefly, PrCa cases (n = 979) and controls (n = 1,251) were recruited from the Ashkenazi Jewish community through letters and advertisements from 1998 through 2005. All men included in this study satisfied the criteria of having both parents of Ashkenazic descent, completed a self-administered epidemiological questionnaire, and provided a DNA sample extracted from mouthwash or blood as previously described [38], [39]. Cases and controls were on average 68 years at participation, and the majority (>75%) of participants had obtained a college or graduate/professional degree (see Table S1). Nearly all cases (95%) and controls (98%) had undergone serum PSA testing or digital rectal examination (DRE) for PrCa screening. Cases were twice as likely as controls to report a first-degree relative with prostate cancer (28% vs. 14%, p<0.0001) [38], [39].

The average age at PrCa diagnosis was 65 years and the majority of cases (85%) were diagnosed because of an abnormal PSA or DRE test. Clinical information on Gleason score, and extent of disease based on tumor invasiveness, tumor present at resection margins, prostate capsule invasion, seminal vesicle involvement, and lymph node involvement was obtained from pathology reports of prostate biopsies or radical prostatectomy tissues; records were available on 92% of the cases. Approximately two thirds of cases had a Gleason score of 2–6, 25% had a Gleason score of 7, and 12% had Gleason score 8–10 (Table S1); approximately half of the cases were classified as having aggressive PrCa [38,39).

### Selection of SNPs and Genotyping Methods

We selected a total of 31 SNPs in different genomic regions based on the cumulative evidence for association with PrCa in multiple large GWAS published reports at the time this study was designed [8]–[20]. These SNPs also included variants that were reported to be associated with aggressive PrCa [21]–[23] and/or serum PSA levels [24], [25]. Detailed information about these SNPs is available from the NCBI dbSNP: http://www.ncbi.nlm.nih.gov/projects/SNP. TaqMan custom genotyping assays (ABI, Foster City, CA, USA) were designed for each SNP and optimized based on concordance with HapMap data. A total of 936 cases and 1,223 controls with sufficient DNA were successfully genotyped for 31 SNPs. The degree of missing genotype data varied across the 31 SNP ranging from 1% to 11% (average 2% for all SNPs). A completion rate threshold of 85% per sample was used as acceptable. For quality control (QC) 21 subjects were genotyped in duplicate and the overall concordance rate was 99.9%.

### Statistical Analysis

#### Individual SNP analysis

The distribution of SNP alleles and genotypes was assessed separately for cases and controls, and deviation of genotype frequencies from Hardy-Weinberg equilibrium (HWE) among controls was assessed by χ2-tests. All SNPs were in HWE. Unconditional logistic regression was used to examine associations between SNPs and PrCa risk and to compute odds ratios (OR) and 95% confidence intervals (CI) [40] for allele-specific and genotype-specific associations. In genotype-level analyses (presented in Table S2) we first examined models where we compared men heterozygous (e.g. CT) and homozygous for the minor allele frequency (e.g. TT) to men homozygous for the major allele frequency (e.g. CC –used as reference), based on the frequency distribution of genotypes in controls. Then we also examined dominant (e.g. TT and CT vs. CC) and recessive (e.g. TT vs. CT and CC) models. Associations between SNPs and PrCa risk were adjusted for age at diagnosis (cases) and age at study participation (controls). Additional adjustment for first-degree family history of PrCa and PSA or DRE screening did not substantially change the ORs estimates for the SNP genotypes, thus the final models presented were adjusted only for age. For our primary analyses using allelic additive models, we used a p = 0.002 (two-sided) to indicate a statistically significant result to account for multiple comparisons of 31 individual SNPs (Bonferroni corrected p-value threshold). A permutation procedure was also used to account for the effect of multiple comparisons of 31 GWAS SNPs [41]. Pairs of case-control labels and ages were permuted in order to approximate the distribution of the age-adjusted p-values under the null hypothesis. Ages and case-control labels were permuted together to preserve any relationship that may exist between age and case-control status and allow age-adjusted p-values to be calculated for each permutation that were consistent with the original analysis. For each permutation, allelic additive models were fit for 31 SNPs. Permutation p-values can be interpreted as the probability of observing a p-value less than or equal to what was observed for a given order statistic under the null hypothesis of no association between PrCa and any of the 31 SNPs [41]. A SNP was considered to be statistically significantly associated with PrCa if the permuted p-value was ≤0.05 (two-sided). We have used this methodology in another paper examining associations between SNPs in DNA repair genes and risk of PrCa accounting for multiple comparisons [42]

We also examined the associations between SNPs and PrCa according to strata defined by Gleason score, and a composite measure of disease severity. For these analyses, prostate cancer cases were grouped into two strata: those with Gleason scores of 2–6 and those with Gleason scores of 7–10. Aggressive prostate cancer was defined as having either a Gleason score 7–10 or at least two of the following characteristics documented on the pathology report: tumor invasiveness, tumor present at resection margins, prostate capsule invasion, seminal vesicle involvement, and/or lymph node involvement. The frequency of SNP alleles (allelic additive model) or genotypes (for genotype-based and dominant or recessive models) in each group of cases (i.e., those with more aggressive vs. less aggressive or those with high (7–10) and low (2–6) Gleason score cancers) were compared to the frequency of alleles/genotypes among controls using polytomous logistic regression models [43]. We also tested for heterogeneity of ORs estimates of SNPs associations between less aggressive vs. more aggressive PrCa and between tumors with a Gleason score 2–6 vs. 7–10 to identify SNPs significantly associated with advanced disease but not with less aggressive cancer and vice versa [40]

Associations between SNPs and PrCa risk were examined in strata defined by age at diagnosis: age ≤60 and >60 years to explore if SNPs were associated with young onset PrCa, as well as by family history of PrCa (yes vs. no), and by family history of other common cancers including lung, colorectal, breast, ovarian and bladder cancers. To test effect modification, interaction terms between SNPs genotypes and age (≤60, >60 years) or family history of cancer (i.e., prostate, lung, colorectal, breast, ovarian or bladder cancers) were included in models containing the main genotype effects in separate logistic regression models. The log likelihood of reduced models with main effects only were compared with the log likelihood of fully saturated models that also contained the interaction terms, using a likelihood ratio test to evaluate the statistical significance of the interaction(s) terms [44]

#### Multiple risk alleles analyses

For the 15 SNPs that were associated with PrCa at a nominal p≤0.05 and two SNPs (rs10934853 and rs9364554) that had p-values of 0.055 and 0.057, respectively from allelic additive models (total 16 autosomal SNPs and 1 SNP on X-chromosome); we calculated the cumulative number of risk alleles that each subject carried by summing over the risk alleles (for the SNPs that were inversely associated with PrCa we used the reference/major allele as the risk allele). We examined the distribution of number of risk alleles between PrCa cases and controls and then created four categories of number of risk alleles by selecting cut-off points based on quartiles of the distribution among the controls. We investigated associations between the cumulative number of risk alleles (both continuous and categorical) and risks of overall PrCa, as well as disease aggressiveness using logistic and polytomous logistic regressions, respectively, adjusting for age. We also examined associations between cumulative number of risk alleles and PrCa in strata defined by age at diagnosis (≤60 vs. >60 years) and by family history of PrCa (yes vs. no). Finally, we calculated the C-statistics (equivalent to the area under the receiver operating characteristic (ROC) curves: AUC) for three logistic regression models: the first model included only the cumulative number of risk alleles, the second one included age (we used age at diagnosis for cases and age at participation for controls) and family history of PrCa; and the third model included the cumulative number of risk alleles plus age and family history of PrCa to evaluate and compare the predictive value of these variables in discriminating individuals with PrCa and without cancer. We compared the AUC curves for all three models for overall risk of PrCa as well as aggressive PrCa phenotype. SAS version 9.2 (SAS Institute, Carry NC) and STATA version 11 (STATA Corporation, College Station, TX) were used for all statistical analyses.

## Results

### Individual SNP Analyses

Table 1 presents associations between 31 SNPs previously identified in PrCa GWAS studies, and overall risk of PrCa in men of Ashkenazic descent using allelic additive model. Overall, 15 SNPs were associated with PrCa at nominal p≤0.05 and of these, 8 SNPs were associated with risk of PrCa at p≤0.002 (corrected p-value threshold for multiple-comparison and presented in bold in Table 1). The permutation procedure adjusting for multiple comparisons yielded the same results showing the same 8 SNPs to be statistically significantly associated with PrCa risk in allelic additive models (permutated p-values ≤0.05). Most of the observed associations were modest with ORs ranging from of 1.22 to 1.42 per risk allele (or ORs of 0.66 to 0.80 for those SNPs inversely associated with PrCa). Results of genotype-level analyses including dominant and recessive models are presented in Table S2.

**Table 1:** Associations of GWAS SNPs with Overall Risk of Prostate Cancer among Ashkenazic Men.

CHROM	dbSNP	Alleles Major/Minor	MAF in Controls	Allelic Additive Model		
				OR*	95% CI	P
2p15	rs721048	G/A	0.134	1.03	0.86–1.25	0.72
2p21	rs1465618	G/A	0.172	1.14	0.96–1.35	0.13
2q31	rs12621278	A/G	0.047	0.91	0.68–1.21	0.52
3p12	rs2660753	C/T	0.219	1.15	0.99–1.33	0.055
3q21	rs10934853	C/A	0.294	0.98	0.86–1.13	0.80
4q22	rs12500426	A/C	0.465	0.91	0.81–1.03	0.15
4q22	rs17021918	C/T	0.339	0.91	0.80–1.04	0.15
4q24	rs7679673	C/A	0.492	0.89	0.79–1.01	0.057
**6q25**	**rs9364554**	**C/T**	**0.172**	**1.29**	**1.11–1.51**	**0.001**
7p15	rs10486567	C/T	0.297	0.93	0.82–1.06	0.29
7q21	rs6465657	T/C	0.422	1.09	0.96–1.23	0.17
8p21	rs1512268	G/A	0.437	1.08	0.95–1.22	0.24
8q24	rs10086908	A/G	0.239	0.95	0.82–1.10	0.50
8q24	rs1447295	C/A	0.067	1.01	0.79–1.30	0.92
8q24	rs16901979	G/T	0.031	1.30	0.94–1.80	0.12
8q24	rs620861	G/A	0.402	0.83	0.73–0.95	0.005
**8q24**	**rs6983267**	**T/G**	**0.477**	**1.34**	**1.19–1.52**	**5.7×10^−7^**
**10q11**	**rs10993994**	**C/T**	**0.489**	**1.22**	**1.08–1.38**	**0.002**
**11q13**	**rs10896438**	**T/G**	**0.248**	**1.26**	**1.09–1.45**	**0.001**
**11q13**	**rs10896449**	**G/A**	**0.346**	**0.80**	**0.70–0.92**	**0.002**
11q13	rs12793759	G/A	0.213	1.17	1.02–1.35	0.03
11p15	rs7127900	C/T	0.248	1.21	1.05–1.40	0.008
17p12	rs4054823	A/G	0.465	1.03	0.92–1.17	0.59
**17q12**	**rs4430796**	**C/T**	**0.432**	**1.25**	**1.10–1.42**	**0.0001**
17q21	rs11649743	C/T	0.142	0.87	0.73–1.05	0.16
17q24	rs1859962	G/T	0.468	0.89	0.78–1.00	0.05
**19q13**	**rs17632542**	**T/C**	**0.079**	**0.66**	**0.52–0.85**	**0.001**
19q13	rs2735839	G/A	0.183	0.85	0.72–1.00	0.05
19q13	rs8102476	C/T	0.389	0.87	0.77–0.99	0.03
22q13	rs5759167	A/C	0.498	1.19	1.05–1.35	0.006
**Xp11**	**rs5945619**	**A/G**	**0.240**	**1.42**	**1.16–1.72**	**0.001**

* ORs and corresponding 95% CI are age-adjusted in all models; MAF = Minor Allele Frequency.

Bold font represent SNPs that show statistically significant associations at α = 0.002 (2-sided; correcting for multiple comparisons (testing of 31 SNPs).

Next, we examined associations between the 31 SNPs and PrCa according to pathologic features of PrCa (less vs. more aggressive cancer) using polytomous logistic regression models adjusted for age (Table 2). In this analysis, two SNPs (rs17632542 at 19q13 and rs5945619 at Xp11) were associated with non-aggressive PrCa; three other SNPs (rs7679673 at 4q24, rs9364554 at 6q25 and rs10993994 at 10q11) were associated with more aggressive cancer and one SNP (rs6983267 at 8q24) was associated with both forms of PrCa using a p = 0.002 as the cutoff point for statistical significance. SNPs that showed statistically significant risks for more aggressive but not for less-aggressive cancer were: rs7679673 (OR = 0.81; 95% CI: 0.69–0.84; p = 0.002), rs9364554 (OR = 1.37; 95% CI 1.13–1.65; p = 0.001) and rs10993994 (OR = 1.26; 95% CI: 1.08–1.47; p = 0.002). SNP rs6983267 at 8q24 was associated with both less and more aggressive PrCa with ORs of 1.31 (p = 0.001) and 1.36 (p<0.0001) per risk allele, respectively. Results were similar when cases were stratified by the Gleason score: 2–6 vs. 7–10 (data not shown).

**Table 2:** Associations of GWAS SNPs with Clinical Characteristics of Prostate Cancer.

CHROM	dbSNP	Alleles Major/Minor	MAF inControls	Non-Aggressive Prostate Cancer			Aggressive Prostate Cancer†		
				OR*	95% CI	P	OR*	95% CI	P
2p15	rs721048	G/A	0.134	0.99	0.78–1.27	0.96	1.08	0.86–1.36	0.51
2p21	rs1465618	G/A	0.172	1.11	0.89–1.39	0.34	1.17	0.94–1.44	0.16
2q31	rs12621278	A/G	0.047	0.78	0.53–1.16	0.22	1.04	0.73–1.47	0.83
3p12	rs2660753	C/T	0.219	1.20	1.00–1.45	0.05	1.14	0.95–1.36	0.17
3q21	rs10934853	C/A	0.294	1.06	0.89–1.26	0.52	0.88	0.74–1.05	0.16
4q22	rs12500426	A/C	0.465	0.92	0.78–1.08	0.30	0.91	0.78–1.06	0.24
4q22	rs17021918	C/T	0.339	0.93	0.78–1.10	0.37	0.88	0.75–1.04	0.15
**4q24**	**rs7679673**	**C/A**	**0.492**	0.95	0.81–1.12	0.57	**0.81**	**0.69–0.94**	**0.002**
**6q25**	**rs9364554**	**C/T**	**0.172**	1.16	0.95–1.42	0.15	**1.37**	**1.13–1.65**	**0.001**
7p15	rs10486567	C/T	0.297	1.00	0.84–1.19	0.97	0.92	0.77–1.09	0.32
7q21	rs6465657	T/C	0.422	1.13	0.96–1.32	0.14	1.05	0.90–1.23	0.50
8p21	rs1512268	G/A	0.437	1.20	1.02–1.42	0.03	0.99	0.84–1.16	0.88
8q24	rs10086908	A/G	0.239	0.90	0.75–1.09	0.30	1.05	0.87–1.25	0.62
8q24	rs1447295	C/A	0.067	1.13	0.83–1.53	0.45	1.00	0.73–1.37	0.99
8q24	rs16901979	G/T	0.031	1.52	1.02–2.26	0.04	1.21	0.80–1.84	0.36
8q24	rs620861	G/A	0.402	0.79	0.67–0.94	0.007	0.85	0.72–1.00	0.05
**8q24**	**rs6983267**	**T/G**	**0.477**	**1.31**	**1.11–1.53**	**0.001**	**1.36**	**1.16–1.58**	**<0.0001**
**10q11**	**rs10993994**	**C/T**	**0.489**	1.16	0.99–1.37	0.07	**1.26**	**1.08–1.47**	**0.002**
11q13	rs10896438	T/G	0.248	1.26	1.05–1.51	0.012	1.26	1.06–1.50	0.009
11q13	rs10896449	G/A	0.346	0.86	0.72–1.02	0.09	0.78	0.66–0.93	0.004
11q13	rs12793759	G/A	0.213	1.23	1.03–1.48	0.03	1.15	0.96–1.38	0.12
11p15	rs7127900	C/T	0.248	1.26	1.05–1.52	0.011	1.19	1.00–1.43	0.05
17p12	rs4054823	A/G	0.465	0.97	0.83–1.14	0.72	1.09	0.94–1.27	0.27
17q12	rs4430796	C/T	0.432	1.26	1.07–1.48	0.005	1.25	1.07–1.47	0.005
17q21	rs11649743	C/T	0.142	0.97	0.77–1.23	0.81	0.77	0.60–0.98	0.03
17q24	rs1859962	G/T	0.468	0.87	0.74–1.02	0.08	0.87	0.74–1.01	0.07
**19q13**	**rs17632542**	**T/C**	**0.079**	**0.53**	**0.37–0.77**	**0.001**	0.72	0.53–0.98	0.04
19q13	rs2735839	G/A	0.183	0.76	0.61–0.95	0.015	0.92	0.75–1.13	0.41
19q13	rs8102476	C/T	0.389	0.83	0.71–0.98	0.03	0.90	0.77–1.06	0.22
22q13	rs5759167	A/C	0.498	1.17	1.00–1.37	0.05	1.22	1.04–1.42	0.013
**Xp11**	**rs5945619**	**A/G**	**0.240**	**1.49**	**1.16–1.91**	**0.002**	1.37	1.07–1.75	0.011

† Aggressive prostate cancer was defined as having either a Gleason score 7 or higher, or at least two of the following characteristics documented on the pathology report: tumor invasiveness, tumor present at resection margins, prostate capsule invasion, seminal vesicle involvement, and lymph node involvement. Prostate cancer cases with missing information on disease pathological characteristics (n = 60) were excluded from these analyses.

* Odds ratios (ORs) and 95% CI for SNP genotypes for non-aggressive vs. aggressive prostate cancer were estimated using polytomous logistic regression models adjusted for age using allelic additive models; Bold font represent SNPs that show statistically significant associations at α = 0.002 (2-sided).

MAF = Minor Allele Frequency.

To explore if any of the SNPs were associated with an early-age at PrCa onset, we examined risk of PrCa in strata defined by age at diagnosis: ≤60 vs. >60 years, and present SNPs that were associated with young onset PrCa: ages ≤60 years (see Table S3). Two SNPs, rs2660753 at 3p12 and rs10896449 at 11q13, were associated with younger age (≤60 years) at PrCa diagnosis, but not with older ages (p-values for interactions were 0.04 and 0.02, respectively). For rs2660753, men aged ≤60 years with the CT and TT genotypes had ORs of 1.46 and 2.48 for PrCa, respectively, in comparison to men with the CC genotype. Whereas in the same age category, for rs10896449 men with the AG and AA genotypes had ORs of 0.68 and 0.33, respectively, in comparison to men with the GG genotype.

We also examined whether risk of PrCa associated with these SNPs varied by family history (FH) of PrCa or FH of other common cancers, i.e., lung, colorectal, breast, ovarian and bladder cancer using information on FH of cancer provided by participants (see Table S4). For first-degree FH of PrCa, SNP rs8102476 at 19q13 showed a potential interaction (p = 0.02), where men with FH of PrCa and CC or CT/TT genotype had ORs of 2.99 (95% CI: 2.12–4.22) and 1.63 (95% CI: 1.20–2.20), respectively, in comparison to men with CC genotype but without FH of PrCa (Table S4a). For FH of other cancers, we predicted that if there was a syndrome-like association, we should see the risk genotype increased in cases with FH of a specific cancer (e.g., lung cancer) compared to controls with no FH of lung cancer, and the association should not be present in PrCa cases with no FH of lung cancer compared to controls with no FH of lung cancer. By contrast, if the risk genotype was associated specifically with PrCa independent of lung cancer, there should be no difference in the association of the risk allele in men with or without FH of lung cancer compared to the controls without a FH of lung cancer. Whereas, if the risk genotype was associated with lung cancer, we should detect an association in controls with a FH of lung cancer vs. controls without a FH of lung cancer as recently proposed by Ghosh et al [45]. Tables S4b and S4c present associations of SNPs with PrCa stratified by any FH of lung cancer or any FH of breast cancer, respectively (there were no statistically significant associations with FH of colon/rectal, ovarian or bladder cancer and therefore those data are not presented). We observed two SNPs that had potential interactions with risks of PrCa and another cancer: rs17021918 at 4q22 and any FH of lung cancer (p for interaction = 0.03), and rs10896449 and any FH of breast cancer (p for interaction = 0.01).

### Multiple Risk Alleles Analysis

**Figure 1:**
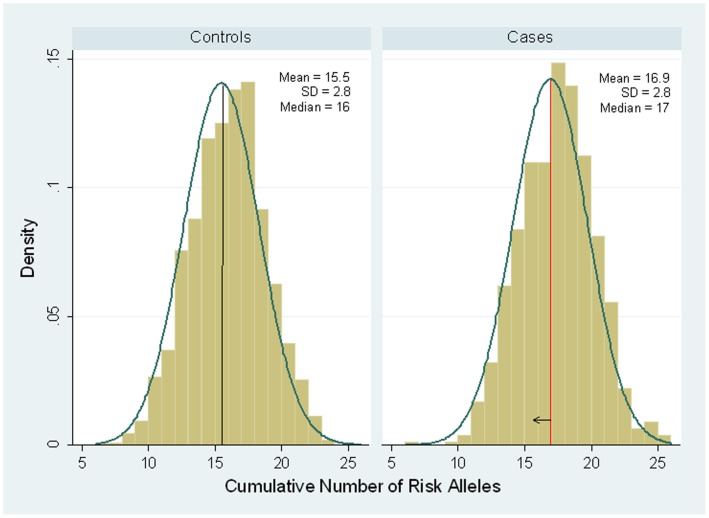
Distribution of the cumulative number of risk alleles among prostate cancer cases and control subjects. Solid lines represent the median number of risk alleles in controls (black line) and cases (red line). The arrow shows the shift in median number of risk alleles between cases and controls. Abbreviation, SD: standard deviation.

Since GWAS SNPs were identified in independent regions of the genome, we were interested in examining risk of PrCa in relation to cumulative numbers of risk alleles that an individual carries. Figure 1 provides the distribution of number of risk alleles among PrCa cases and controls. Cases carried on average an additional risk allele in comparison to controls (median of 17 vs. 16 risk alleles in cases and controls, respectively; p<0.0001). There was an increasing risk of PrCa with increasing quartiles of cumulative number of risk alleles (Table 3; p for trend <0.0001), where men in the highest quartile had an OR of 3.70 (95% CI: 2.76–4.97) for PrCa in comparison to those in the lowest quartile. However, men in the highest vs. lowest quartile of number of risk alleles had similar ORs for non-aggressive PrCa (OR = 3.84; 95% CI: 2.60–5.69) vs. aggressive cancer (OR = 3.76; 95% CI: 2.57–5.50), respectively. When data were stratified by age at PrCa diagnosis, the average number of cumulative risk alleles was slightly higher among cases diagnosed at age 60 years or younger (17.3 risk alleles) in comparison to cases diagnosed at age >60 years (16.8 risk alleles); however the number of risk alleles for both case groups was higher in comparison to controls (the average number of risk alleles was 15.5 in controls both aged ≤60 and >60 years at participation). Table 3 presents results stratified by age at diagnosis and as observed men aged ≤60 years at diagnosis had an OR of 5.20 (95% CI: 2.94–9.19) for PrCa when comparing the highest vs. lowest quartile; whereas among men aged >60 years there was an OR = 3.30 (95% CI 2.32–4.68). Interestingly, ORs were similar when comparing highest vs. lowest quartile of number of risk alleles in the stratified analysis by first-degree family history of PrCa (Table 3).

**Table 3:** Associations of cumulative number of risk alleles with overall prostate cancer, and stratified by clinical features, age at diagnosis and family history of prostate cancer.

Overall Risk of Prostate Cancer	Quartiles* (Q) of Cumulative Number of Risk Alleles				P for trend
	Q1 (8–13)*	Q2 (14–15)*	Q3 (16–17)*	Q4 (18–24)*	
Controls (n = 1,056); n (%)	257 (24.3)	258 (24.4)	295 (27.9)	226 (23.3)	
Cases (n = 774); n (%)	91 (11.8)	150 (19.4)	200 (25.8)	333 (43.0)	
OR† (95% CI)	1.00	1.68 (1.22–2.30)	1.89 (1.40–2.56)	3.70 (2.76–4.97)	<0.0001
Clinical Features of Prostate Cancer					
Controls (n = 1,056); n (%)	257 (24.3)	258 (24.4)	295 (27.9)	226 (23.3)	
Non-Aggressive Cases (n = 344); n (%)	40 (11.6)	74 (21.5)	78 (22.7)	152 (44.2)	
OR‡ (95% CI)	1.00	1.86 (1.22–2.85)	1.67 (1.10–2.53)	3.84 (2.60–5.69)	<0.0001
Aggressive Cases (n = 383); n (%)	44 (11.5)	63 (16.5)	111 (29.0)	165 (43.1)	
OR‡ (95% CI)	1.00	1.44 (0.94–2.20)	2.15 (1.45–3.18)	3.76 (2.57–5.50)	<0.0001
Age ≤60 years at Prostate Cancer Diagnosis					
Controls (n = 272); n (%)	65 (23.9)	67 (24.6)	74 (27.2)	66 (24.3)	
Cases (n = 238); n (%)	22 (9.2)	38 (16.0)	62 (26.1)	116 (48.7)	
OR† (95% CI)	1.00	1.67 (0.89–3.13)	2.47 (1.37–4.46)	5.20 (2.94–9.19)	<0.0001
Age >60 years at Prostate Cancer Diagnosis					
Controls (n = 784); n (%)	192 (24.5)	191 (24.4)	221 (28.2)	180 (23.0)	
Cases (n = 536); n (%)	69 (12.9)	112 (20.9)	138 (25.8)	217 (40.5)	
OR† (95% CI)	1.00	1.76 (1.21–2.54)	1.73 (1.21–2.47)	3.30 (2.32–4.68)	0.001
No First-Degree Family History of Prostate Cancer					
Controls (n = 906); n (%)	228 (25.2)	216 (23.8)	260 (28.7)	202 (22.3)	
Cases (n = 546); n (%)	70 (12.8)	118 (21.6)	137 (25.1)	221 (40.5)	
OR† (95% CI)	1.00	1.84 (1.29–2.63)	1.71 (1.21–2.41)	3.42 (2.45–4.77)	<0.0001
Positive First-Degree Family History of Prostate Cancer					
Controls (n = 150); n (%)	29 (19.3)	42 (28.0)	35 (23.3)	44 (29.3)	
Cases (n = 228); n (%)	21 (9.2)	32 (14.0)	63 (27.6)	112 (49.1)	
OR† (95% CI)	1.00	1.04 (0.50–2.16)	2.43 (1.21–4.90)	3.51 (1.81–6.81)	<0.0001

* The cutoff points for quartiles were determined based on the distribution of number of risk alleles among all controls; numbers in parenthesis represent the range of number of risk alleles for each quartile.

Percentages represent row percents.

† ORs and 95% CI were computed using logistic regression models adjusted for age; cases or controls with missing SNPs genotype data were excluded.

‡ ORs and 95% CI for clinical features of prostate cancer were computed using polytomous logistic regression adjusted for age. Cases or controls with missing SNPs genotype data or cases with missing clinical information for prostate cancer (n = 60) were excluded.

**Figure 2:**
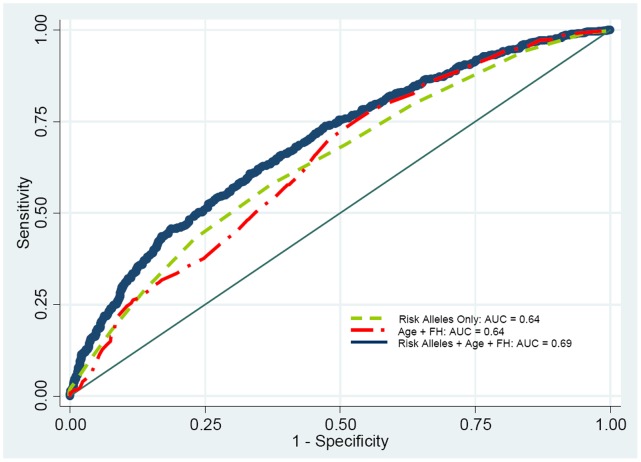
Receiver operating characteristic (ROC) curves for risk prediction of prostate cancer for three different models incorporating cumulative number of risk alleles, age at diagnosis and family history (FH) of prostate cancer.

Finally we compared the predictive ability of age at diagnosis, family history of PrCa and cumulative number of risk alleles in discriminating patients with PrCa vs. controls, as well as in predicting risk of more aggressive cancer. We fitted three separate logistic regression models for both outcomes: i.e. overall PrCa as well as aggressive cancer; the first model included only the cumulative number of risk alleles, the second one contained age and family history of PrCa, and the third model included the cumulative number of risk alleles plus age and family history of PrCa. Figure 2 shows the ROC curves for these three models for overall risk of PrCa. Interestingly, the AUC for overall PrCa risk for the model that included only the number of risk alleles compared to one that included age at diagnosis and family history of PrCa resulted in a similar value of 0.64 (Figure 2). The addition of number of risk alleles to the model containing age at diagnosis and family history (FH) of PrCa slightly improved the predictive value for overall PrCa (the AUC increased from 0.64 to 0.69, respectively). Results for aggressive PrCa were similar to those observed for overall risk of PrCa; the AUC for aggressive PrCa slightly increased from 0.66 to 0.71 after adding the cumulative number of risk alleles to the model containing age and FH of PrCa (data not shown).

## Discussion

In this study, we examined associations between 31 SNPs identified by previous GWAS of PrCa in a large case-control study of men of Ashkenazic Jewish descent. Overall, 8 SNPs showed associations with PrCa at p≤0.002 after adjustment for multiple comparisons for 31 independent tests. Most of the observed associations between the GWAS SNPs and PrCa were modest (ORs between 1.22 and 1.42) as previously reported in other GWAS of PrCa [8]–[20]. Moreover, when we examined the association between cumulative number of risk alleles and PrCa we observed an OR of 3.70 (95% CI 2.76–4.97) for PrCa comparing men in the highest vs. lowest quartile. To our knowledge, only the NCI Breast and Prostate Cancer Cohort Consortium conducted a similar analysis and they reported an OR of 5.55 (95% CI 4.85–6.35) for PrCa when comparing highest to lowest deciles for number of risk alleles in more than 10,000 cases and controls [46]. Although our sample size was smaller, our results however, are similar to the NCI Cohort Consortium study indicating that the higher the number the risk alleles a man carriers, the higher the risk. However, the addition of number of risk alleles to the model containing age at diagnosis and family history of PrCa improved slightly the predictive value (the AUC increased from 0.64 to 0.69 for overall PrCa; and from 0.66 to 0.71 for aggressive PrCa) in our study. This indicates that the clinical utility of these SNPs as predictors of PrCa is limited at the moment, although further consideration is required for stratification of men for screening purposes. Nevertheless, genetic markers of disease can be identified at birth; whereas, other risk stratification variables such as the number of affected relatives requires aging of family members, since PrCa is a disease with late age at onset (average age of 70 years). This may provide a window of opportunity for prevention as our knowledge of the natural history and pathogenesis of PrCa improves.

With respect to Ashkenazic populations, one recent study investigated the associations between 29 GWAS SNPs and risk of PrCa among 963 cases, mostly from Memorial Sloan Kettering Cancer Center (MSKCC), and a set of 613 controls from MSKCC, and 1,241 additional controls from New York and Israel [32]. Among participants with complete genotyping data (875 cases and 1,810 controls) nine SNPs were reported to be associated with PrCa risk in age-adjusted models at a nominal p<0.05, while only three SNPs (rs4242382, rs7931342 and rs10896449) remained statistically significant after adjusting for a false discovery rate. Taken together, this previous report [32] and our study genotyped 29 and 31 SNPs, respectively, however, only 12 SNPs overlapped between the two studies. We didn’t genotype SNPs rs4242382 (8q24) and rs7931342 (11q13) in our study population; nevertheless, their result for rs10896449 at 11q13 (OR = 0.80; 95% CI 0.68–0.93; p = 0.005) was similar to ours and confirmed the significance of this SNP risk allele for PrCa. For rs6983267 at 8q24 we report an OR = 1.34 (p-value = 5.7×10^−7^) associated with the G allele in comparison to the T allele, whereas Vijai et al. [32], reported an inverse association based on using a different reference allele: OR = 0.83 for T vs. G allele (p = 0.018). However the inverse association is due only to a different reference comparison group in that study [32] and not due to differences in allele frequencies (the frequency of the T allele was similar in both studies: 52% and 50%, and is similar to the frequency in the Caucasian population of 51%). Vijai et al [32] did not examine risk by pathologic features of PrCa, by age at PrCa diagnosis or by family history of PrCa or other cancers. No other large studies of GWAS SNPs and PrCa have been conducted in Ashkenazim populations, and this is the first study to recruit a comparable control group allowing for various covariate analyses.

We did not observe large heterogeneities in SNP-associations between less aggressive and more aggressive PrCa or by Gleason score (2–6 vs. 7–10). Three SNPs: rs7679673 at 4q24 (OR = 0.81; p = 0.002), rs9364554 at 6q25 (OR = 1.37; p = 0.001) and rs10993994 at 10q11 (OR = 1.26; p = 0.002) were associated with more aggressive cancer, but not less aggressive disease, using a p = 0.002 as the cutoff point for statistical significance. Fewer studies have reported associations with aggressive PrCa and results have been inconsistent [21]–[23], [47], [48]. In a large study of men of European descent from the US and Sweden, Xu and colleagues [22] reported an OR of 1.13 (95% CI: 1.08–1.19; p = 2.1×10^−6^) for rs4054823 at 17p12 and aggressive PrCa. By contrast, among Ashkenazi Jewish men, we did not observe an association between this SNP and risk of overall PrCa (OR = 1.03; 95% CI: 0.92–1.17; p = 0.59) or more aggressive cancer (OR = 1.09; 95% CI: 0.94–1.27; p = 0.27). Ahn and colleagues [23] recently reported three SNPs: rs10993994 in 10q11 (RR = 1.24, 95% CI 1.05–1.48), rs4242382 in 8q24 (RR = 1.40, 95% CI 1.13–1.75), and rs6983267 in 8q24 (RR = 0.67, 95% CI 0.50–0.89) that were associated with risk for metastatic PrCa. In the current analysis, the association between rs10993994 at 10q11 and aggressive cancer (OR = 1.26; p = 0.002) was also observed. However, for rs6983267 at 8q24 we report associations both with less aggressive (ORs = 1.31; p = 0.001) and more aggressive PrCa (OR = 1.36; p<0.0001). It should also be noted that in the current study population the G allele of rs6983267 was the minor frequency allele, whereas in the Ahn et al. [23] study, the T allele was the minor allele; and therefore they reported an inverse association with this SNP and metastatic PrCa [23]. We did not genotype rs4242382 in 8q24, rs1571801 in *DAB21P* gene or rs6497287 in 15q13, which were associated with aggressive PrCa phenotype in other studies [21], [23], [48]. In relation to cumulative risk alleles we reported a similar association between more aggressive and less aggressive PrCa phenotype with ORs of 3.76 and 3.84, respectively, when comparing highest vs. lowest quartile of number of risk alleles. By contrast, the NCI Breast and Prostate Cancer Cohort Consortium study reported a stronger association for number of risk alleles for localized cases (OR = 6.12) then aggressive cases (OR = 4.35) when comparing men in the highest vs. lowest decile [46]. However differences could be due to study populations, the SNPs that were included in the calculation of cumulative risk alleles, as well as various definitions of more vs. less aggressive PrCa across different studies.

Age at diagnosis and family history of PrCa are two major risk factors that provide risk stratification and are clinical indications for early screening. These risk factors are related, at least in part to genetic susceptibility of PrCa, but complete understanding of the molecular mechanisms responsible for familial clustering and age at onset of this cancer still remains enigmatic. We did not observe large variations in risk by age at diagnosis in our population. Only two SNPs, rs2660753 at 3p12 and rs10896449 at 11q13, were associated with younger age (≤60 years) at PrCa diagnosis, but not with older ages (p-values for interaction were 0.04 and 0.02, respectively). In the younger age group, rs2660753 (3p12) was associated with increased risk of PrCa (ORs of 1.46 and 2.48 when comparing men with the CT or TT genotypes vs. those with the CC genotype). Whereas, for rs10896449 (11q13) there was an inverse association (OR = 0.33; 95% CI: 0.18–0.60) when comparing men homozygous for the minor vs. major alleles. Some studies consider younger age at onset to be <55 years. However, in our study population there were few men diagnosed at age 55 years or younger and thus, we had limited statistical power to examine PrCa risk in this age stratum. Interestingly, for cumulative number of risk alleles among men diagnosed at younger ages (≤60 years), we report a higher OR of 5.20 (95% CI: 2.94–9.19) for PrCa for highest vs. lowest category of risk alleles in comparison to older ages (>60 years) at diagnosis. Our result is consistent with the NCI Cohort Consortium study, which also reported a stronger association for cases diagnosed at ages <65 years (OR = 7.21,95% CI: 5.66–9.18) [46], and indicates that GWAS SNPs have better predictive ability for PrCa among younger men, and thus could be useful in screening younger men at heightened risk.

In relation to family history of PrCa, our results are consistent with previous GWAS studies demonstrating that these SNPs do not explain the majority of the familial/hereditary risk component of this cancer [26]–[28]. The only SNP that showed a suggestive influence of a family history of PrCa in our dataset was rs8102476 at 19q13, where men with family history of PrCa and CC or CT/TT genotype had ORs of 2.99 (95% CI: 2.12–4.22) and 1.63 (95% CI: 1.20–2.20), respectively, in comparison to men with CC genotype, but without family history of PrCa (p for interaction = 0.02). In addition, ORs for comparison of highest vs. lowest quartile of number of risk alleles were similar in the stratified analysis by first-degree family history of PrCa (ORs of 3.42 vs. 3.51, respectively), which indicates that these GWAS SNPs do not explain the risk associated with FH of PrCa in our sample set.

In this study, we also examined whether associations between SNPs and PrCa varied by family history of other cancers. This was an exploratory analysis to examine whether there is any suggestive evidence of segregation of PrCa and other cancers that could be explained by GWAS SNPs. We observed interactions between PrCa and rs17021918 (4q22) and any FH of lung cancer (p = 0.03), and between any FH of breast cancer and rs10896449 at 11q13 (p = 0.01). Our study is one of the first investigations to indicate that the above SNPs identified through GWAS of PrCa also segregates with risks of PrCa and lung or breast cancers in families. Another recent report also suggested that PrCa risk alleles could be associated with other malignancies including melanoma and hematopoietic cancers [49]. However, these results should be interpreted with caution as there was limited statistical power to assess interactions of GWAS SNPs with familial history of other cancers (especially if they are rare) and therefore such observations will need to be confirmed in larger datasets.

The analyses investigating the risk based on total number of SNP risk alleles provided an interesting display of a shift in the normal distribution of risk alleles between cases and controls. This pattern of risk allele distribution has been associated with conditions that have a normal distribution in the population (e.g., height) [50], and has been proposed as a polygenetic risk model that affects risk of breast and prostate cancers [51]. We speculate that perhaps PrCa and other cancers showing a similar distribution and shift in the number of risk alleles represent phenotypes that may have the characteristics of a normally distributed trait and that with ageing nearly all men could be diagnosed with PrCa, whereas an increased number of risk alleles might affect the age distribution of diagnosis. To this point, we observed a greater number of total risk alleles for men with early onset (age at diagnosis) PrCa, suggesting that this characteristic is associated with a shift in the timing of the manifestation and/or clinical detection of PrCa. Understanding the distribution of risk alleles and their significance will require additional analyses from other studies and a deeper understanding of the PrCa phenotype and pathogenesis.

Our study has strengths and limitations. With a sample size of 1,800 Ashkenazi Jewish men, our study was able to detect ORs of 1.35 or higher for overall PrCa risk for SNPs with minor allele frequency (MAF) ≥20%, using 80% statistical power, a log-additive model, and type I error α = 0.002 (based on Bonferroni correction for 31 independent tests). Men were recruited using a novel strategy of enrollment by advertisement and were requested to provide all materials through the mail. As presented in Table S1, over 75% of the study population had at least a college degree that facilitated the completion of the self-administered questionnaire and the self-obtained DNA sample. We obtained detailed information on family history of prostate cancer and other cancers using a self-administered questionnaire and men provided their own pathology reports, significantly reducing the labor involved in obtaining medical records. Nevertheless, such a recruitment strategy has the potential to introduce bias into the study sample. Therefore, these data should be interpreted in light of this recruitment strategy. Despite this fact, the prevalence of GWAS SNP alleles in our study is similar to other large GWAS studies, providing assurance that potential selection bias is minimized. Since only 1% of cases and controls were <50 year old, we had limited power to examine associations with very early ages at diagnosis and thus, our findings are relevant to men diagnosed with this disease at age ≥50 years. The study also had limited statistical power to assess interactions between the SNPs and family history of other cancers, and thus some of the observed association and interactions are mainly suggestive and should be interpreted with caution. Larger studies will be needed to confirm these potential interactions of SNPs by family history of other cancers.

In conclusion, we report that a subset of PrCa risk loci previously identified through GWAS in men of European ancestry are associated with overall risk of PrCa in men of Ashkenazic descent. However, since not all the SNPs were associated with PrCa, our findings indicate possible genetic differences between populations of European and Ashkenazic ancestry with regard to genetic susceptibility of PrCa. Consistent with previous findings, our results also suggest that these risk variants do not explain the majority of risk associated with aggressive disease or family history of PrCa. However, the cumulative number of risk alleles for GWAS SNPs could help identify men at heightened risk for younger age at PrCa onset. Since genetic risk can be ascertained long before PrCa incidence and/or family history of this cancer is recognized, it may provide an opportunity to screen and/or intervene once there is sufficient knowledge of the natural history of this disease. This process will entail a broader discussion of risk/benefits of genetic screening between patients and physicians in clinical practice. With current uncertainties over whether to use serum PSA as a screening tool for prostate cancer to prevent mortality from this disease, additional population risk stratification is needed and genetic tests might become useful as more variants are discovered.

## Supporting Information

Table S1 Selected characteristics of Ashkenazi Jewish prostate cancer cases and controls.
(DOC)

Table S2 Associations of GWAS SNPs with Overall Risk of Prostate Cancer among Ashkenazic Men (Genotype Risk Models).
(DOC)

Table S3 Associations of GWAS SNPs with Young Onset Prostate Cancer.
(DOC)

Table S4 Associations of GWAS SNPs with Risk of Prostate Cancer stratified by Family History of Prostate Cancer and Other Cancers.
(DOC)
