# Supplementary material for: Characterization of SNPs Associated with Prostate Cancer in Men of Ashkenazic Descent from the Set of GWAS Identified SNPs: Impact of Cancer Family History and Cumulative SNP Risk Prediction
Source: PLoS One. 2013 Apr 3;8(4):e60083. doi: 10.1371/journal.pone.0060083 (PMC3616024; doi:10.1371/journal.pone.0060083)
Supplement: Table S1 — Selected characteristics of Ashkenazi Jewish prostate cancer cases and controls. (DOC) [file pone.0060083.s001.doc]

**Supplemental Table S1. Selected characteristics of Ashkenazi Jewish prostate cancer cases and controls**

| **Characteristics** | **Cases**  **(n = 979)** | | **Controls**  **(n = 1,251)** | | **P*** |
| --- | --- | --- | --- | --- | --- |
| Age at participation (years) | n | % | n | % | 0.01 |
| < 50 | 11 | 1.1 | 12 | 1.0 |  |
| 50 - 59 | 146 | 14.9 | 284 | 22.7 |  |
| 60 - 69 | 345 | 35.2 | 382 | 30.5 |  |
| 70 - 79 | 365 | 37.3 | 427 | 34.1 |  |
| ≥ 80 | 112 | 11.4 | 146 | 11.7 |  |
| First-degree family history of prostate cancer |  |  |  |  | <0.0001 |
| Yes | 276 | 28.2 | 179 | 14.3 |  |
| No | 703 | 71.8 | 1072 | 85.7 |  |
| Smoking status |  |  |  |  | 0.93 |
| Non smoker | 427 | 43.6 | 543 | 43.4 |  |
| Former smoker | 523 | 53.4 | 668 | 53.4 |  |
| Current smoker | 24 | 2.5 | 33 | 2.6 |  |
| Missing | 5 | 0.5 | 7 | 0.6 |  |
| Education |  |  |  |  | 0.90 |
| High school or less | 63 | 6.4 | 86 | 6.9 |  |
| Post secondary / Some college | 155 | 15.8 | 193 | 15.4 |  |
| College degree | 259 | 26.5 | 320 | 25.6 |  |
| Graduate / Professional degree | 493 | 50.4 | 645 | 51.5 |  |
| Missing | 9 | 0.9 | 7 | 0.6 |  |
| History of BPH |  |  |  |  | <0.0001 |
| Yes | 323 | 33.0 | 538 | 43.1 |  |
| No | 616 | 62.9 | 682 | 54.5 |  |
| Unknown | 40 | 4.1 | 31 | 2.5 |  |
| Age at prostate cancer diagnosis (years) |  |  |  |  |  |
| < 50 | 27 | 2.8 |  |  |  |
| 50 - 59 | 242 | 24.7 |  |  |  |
| 60 - 69 | 443 | 45.3 |  |  |  |
| 70 - 79 | 234 | 23.9 |  |  |  |
| ≥ 80 | 33 | 3.4 |  |  |  |
| Reason for prostate cancer diagnosis |  |  |  |  |  |
| Abnormal PSA | 741 | 73.1 |  |  |  |
| Abnormal DRE | 123 | 12.1 |  |  |  |
| Symptoms | 25 | 2.5 |  |  |  |
| TURP for BPH | 15 | 1.5 |  |  |  |
| Other procedures | 57 | 5.6 |  |  |  |
| Unknown | 18 | 1.8 |  |  |  |
| Gleason score |  |  |  |  |  |
| 2 - 4 | 88 | 9.0 |  |  |  |
| 5 - 6 | 456 | 46.6 |  |  |  |
| 7 | 243 | 24.8 |  |  |  |
| 8 - 10 | 115 | 11.7 |  |  |  |
| Missing | 77 | 7.9 |  |  |  |
| Advanced prostate cancer ^ξ^ |  |  |  |  |  |
| Yes | 484 | 49.4 |  |  |  |
| No | 435 | 44.4 |  |  |  |
| Missing | 60 | 6.1 |  |  |  |

* Chi-square p-value

^ξ^ Advanced prostate cancer was characterized as having either a Gleason score ≥ 7, or at least two of the following characteristics documented on the pathology report: tumor invasiveness, tumor present at resection margins, prostate capsule invasion, seminal vesicle involvement or lymph node involvement

Abbreviations: BPH – Benign prostatic hyperplasia; PSA – Prostate specific antigen; DRE – Digital rectal examination;

TURP – Transurethral resection of the prostate
