# Supplementary material for: Characterization of SNPs Associated with Prostate Cancer in Men of Ashkenazic Descent from the Set of GWAS Identified SNPs: Impact of Cancer Family History and Cumulative SNP Risk Prediction
Source: PLoS One. 2013 Apr 3;8(4):e60083. doi: 10.1371/journal.pone.0060083 (PMC3616024; doi:10.1371/journal.pone.0060083)
Supplement: Table S2 — Associations of GWAS SNPs with Overall Risk of Prostate Cancer among Ashkenazic Men (Genotype Risk Models). (DOC) [file pone.0060083.s002.doc]

**Supplemental Table S2. Associations of GWAS SNPs with Overall Risk of Prostate Cancer among Ashkenazic Men (Genotype Risk Models)**

| CHROM | dbSNP | Alleles  Major / Minor | MAF in  Controls | Heterozygous | | Homozygous | | P-value  Wald (2df) | Dominant Model | | | Recessive Model | | |
| --- | --- | --- | --- | --- | --- | --- | --- | --- | --- | --- | --- | --- | --- | --- |
|  |  |  |  | OR* | 95% CI | OR* | 95% CI |  | OR* | 95% CI | P | OR* | 95% CI | P |
| 2p15 | rs721048 | G / A | 0.134 | 1.09 | 0.89 – 1.34 | 0.59 | 0.24 - 1.47 | 0.34 | 1.07 | 0.88 – 1.30 | 0.51 | 0.57 | 0.23– 1.43 | 0.24 |
| 2p21 | rs1465618 | G / A | 0.172 | 0.98 | 0.80 – 1.20 | 2.18 | 1.29 – 3.67 | 0.012 | 1.06 | 0.87 – 1.29 | 0.55 | 2.19 | 1.30- 3.68 | 0.003 |
| 2q31 | rs12621278 | A / G | 0.047 | 0.91 | 0.67 – 1.23 | 0.88 | 0.15 – 5.31 | 0.81 | 0.91 | 0.67 – 1.22 | 0.52 | 0.89 | 0.15- 5.35 | 0.90 |
| 3p12 | rs2660753 | C / T | 0.219 | 1.20 | 1.00 – 1.44 | 1.20 | 0.82 – 1.77 | 0.10 | 1.20 | 1.00 – 1.43 | 0.04 | 1.12 | 0.77- 1.64 | 0.56 |
| 3q21 | rs10934853 | C / A | 0.294 | 0.99 | 0.82 – 1.18 | 0.96 | 0.69 – 1.33 | 0.97 | 0.98 | 0.83 – 1.17 | 0.84 | 0.97 | 0.70- 1.33 | 0.83 |
| 4q22 | rs12500426 | A / C | 0.465 | 0.93 | 0.76 – 1.14 | 0.83 | 0.65 – 1.07 | 0.35 | 0.90 | 0.75 – 1.09 | 0.28 | 0.87 | 0.70- 1.08 | 0.20 |
| 4q22 | rs17021918 | C / T | 0.339 | 0.81 | 0.67 – 0.97 | 0.93 | 0.69 – 1.24 | 0.08 | 0.83 | 0.70 – 0.99 | 0.04 | 1.03 | 0.78- 1.35 | 0.85 |
| 4q24 | rs7679673 | C / A | 0.492 | 0.84 | 0.68 – 1.03 | 0.80 | 0.62 – 1.02 | 0.14 | 0.83 | 0.68 – 1.00 | 0.05 | 0.89 | 0.72- 1.10 | 0.28 |
| 6q25 | rs9364554 | C / T | 0.172 | 1.47 | 1.21 – 1.78 | 1.16 | 0.74 – 1.82 | 0.0005 | 1.43 | 1.19 – 1.72 | 0.0002 | 1.02 | 0.65- 1.61 | 0.92 |
| 7p15 | rs10486567 | C / T | 0.297 | 1.05 | 0.87 – 1.26 | 0.74 | 0.53 – 1.02 | 0.11 | 0.98 | 0.83 – 1.17 | 0.85 | 0.72 | 0.53- 0.98 | 0.04 |
| 7q21 | rs6465657 | T / C | 0.422 | 1.20 | 0.99 – 1.47 | 1.15 | 0.90 – 1.48 | 0.18 | 1.19 | 0.99 – 1.43 | 0.07 | 1.04 | 0.83- 1.29 | 0.77 |
| 8p21 | rs1512268 | G / A | 0.437 | 1.27 | 1.03 – 1.55 | 1.12 | 0.86 – 1.44 | 0.07 | 1.22 | 1.01 – 1.48 | 0.04 | 0.96 | 0.77- 1.20 | 0.72 |
| 8q24 | rs10086908 | A / G | 0.239 | 1.01 | 0.84 – 1.22 | 0.79 | 0.54 – 1.16 | 0.45 | 0.98 | 0.82 – 1.17 | 0.82 | 0.78 | 0.54- 1.15 | 0.21 |
| 8q24 | rs1447295 | C / A | 0.067 | 1.04 | 0.80 – 1.35 | 0.61 | 0.12 – 3.22 | 0.81 | 1.03 | 0.80 – 1.33 | 0.84 | 0.61 | 0.12- 3.20 | 0.56 |
| 8q24 | rs16901979 | G / T | 0.031 | 1.39 | 0.98 – 1.96 | 0.51 | 0.05 – 4.98 | 0.15 | 1.35 | 0.96 – 1.91 | 0.08 | 0.50 | 0.05- 4.87 | 0.55 |
| 8q24 | rs620861 | G / A | 0.402 | 0.83 | 0.69 – 1.01 | 0.69 | 0.53 – 0.91 | 0.019 | 0.80 | 0.67 – 0.95 | 0.014 | 0.77 | 0.60- 0.98 | 0.04 |
| 8q24 | rs6983267 | T / G | 0.477 | 1.08 | 0.87 – 1.35 | 1.78 | 1.39 – 2.27 | 5.7 e^-7^ | 1.29 | 1.05 – 1.59 | 0.014 | 1.68 | 1.38- 2.05 | <0.0001 |
| 10q11 | rs10993994 | C / T | 0.489 | 1.19 | 0.95 – 1.48 | 1.48 | 1.16 – 1.90 | 0.007 | 1.28 | 1.04 – 1.58 | 0.018 | 1.32 | 1.08- 1.61 | 0.006 |
| 11q13 | rs10896438 | T / G | 0.248 | 1.34 | 1.12 – 1.61 | 1.40 | 0.98 – 1.99 | 0.004 | 1.35 | 1.13 – 1.61 | 0.001 | 1.23 | 0.87- 1.74 | 0.23 |
| 11q13 | rs10896449 | G / A | 0.346 | 0.80 | 0.67 – 0.97 | 0.65 | 0.48 – 0.89 | 0.007 | 0.77 | 0.65 – 0.92 | 0.004 | 0.73 | 0.54- 0.97 | 0.03 |
| 11q13 | rs12793759 | G / A | 0.213 | 1.23 | 1.02 – 1.48 | 1.25 | 0.86 – 1.83 | 0.07 | 1.23 | 1.03 – 1.47 | 0.02 | 1.16 | 0.80- 1.68 | 0.42 |
| 11p15 | rs7127900 | C / T | 0.248 | 1.16 | 0.97 – 1.40 | 1.59 | 1.11 – 2.28 | 0.024 | 1.22 | 1.02 – 1.45 | 0.03 | 1.49 | 1.05- 2.12 | 0.03 |
| 17p12 | rs4054823 | A / G | 0.465 | 1.07 | 0.87 – 1.31 | 1.07 | 0.83 – 1.36 | 0.80 | 1.07 | 0.88 – 1.29 | 0.51 | 1.02 | 0.83- 1.26 | 0.83 |
| 17q21 | rs11649743 | C / T | 0.432 | 0.94 | 0.77 – 1.15 | 0.44 | 0.20 – 1.01 | 0.13 | 0.90 | 0.74 – 1.10 | 0.32 | 0.45 | 0.20- 1.01 | 0.06 |
| 17q21 | rs4430796 | C / T | 0.142 | 1.16 | 0.95 – 1.43 | 1.58 | 1.23 – 2.03 | 0.001 | 1.27 | 1.05 – 1.54 | 0.015 | 1.44 | 1.16- 1.78 | 0.001 |
| 17q24 | rs1859962 | G / T | 0.468 | 0.91 | 0.75 – 1.12 | 0.78 | 0.61 – 1.01 | 0.14 | 0.87 | 0.72 – 1.05 | 0.15 | 0.82 | 0.66- 1.02 | 0.07 |
| 19q13 | rs17632542 | T / C | 0.079 | 0.70 | 0.53 – 0.91 | 0.27 | 0.08 – 0.98 | 0.005 | 0.67 | 0.51 – 0.87 | 0.003 | 0.27 | 0.08- 1.03 | 0.06 |
| 19q13 | rs2735839 | G / A | 0.183 | 0.85 | 0.70 – 1.04 | 0.72 | 0.43 – 1.22 | 0.16 | 0.84 | 0.70 – 1.01 | 0.07 | 0.75 | 0.45- 1.27 | 0.29 |
| 19q13 | rs8102476 | C / T | 0.389 | 0.81 | 0.67 – 0.98 | 0.80 | 0.61 – 1.04 | 0.06 | 0.81 | 0.68 – 0.97 | 0.02 | 0.89 | 0.69- 1.14 | 0.35 |
| 22q13 | rs5759167 | A / C | 0.498 | 1.30 | 1.04 – 1.62 | 1.43 | 1.11 – 1.83 | 0.015 | 1.34 | 1.09 – 1.65 | 0.006 | 1.20 | 0.98- 1.45 | 0.07 |
| Xp11 | rs5945619 | A / G | 0.240 | - | - | 1.42 | 1.16 – 1.72 | 0.001 | - | - | - | 1.42 | 1.16 – 1.72 | 0.001 |

*ORs and corresponding 95% CI are adjusted for age; In this model we compared men heterozygous and homozygous for the minor allele frequency (MAF) to men homozygous for the major allele frequency (used as reference group). MAF = Minor Allele Frequency.
