# Supplementary material for: Characterization of SNPs Associated with Prostate Cancer in Men of Ashkenazic Descent from the Set of GWAS Identified SNPs: Impact of Cancer Family History and Cumulative SNP Risk Prediction
Source: PLoS One. 2013 Apr 3;8(4):e60083. doi: 10.1371/journal.pone.0060083 (PMC3616024; doi:10.1371/journal.pone.0060083)
Supplement: Table S3 — Associations of GWAS SNPs with Young Onset Prostate Cancer. (DOC) [file pone.0060083.s003.doc]

**Supplemental Table S3. Associations of GWAS SNPs with Young Onset Prostate Cancer ***

| Chrom | dbSNP* | Genotype | Age ­≤ 60 | Age > 60 | P for  Interaction |
| --- | --- | --- | --- | --- | --- |
|  |  |  | Cases (%) / Controls (%)  OR (95% CI) | Cases (%) / Controls (%)  OR (95% CI) |  |
| **3p12** | **rs2660753** | CC | 149 (52.1) / 200 (63.1)  1.00 (ref) | 371 (58.7) / 545 (60.6)  1.00 (ref) |  |
|  |  | CT | 113 (39.5) / 104 (32.8)  **1.46 (1.03 – 2.05)** | 231 (36.7) / 304 (33.8)  1.12 (0.90 – 1.39) | **0.04** |
|  |  | TT | 24 (8.4) / 13 (4.1)  **2.48 (1.22 – 5.03)** | 30 (4.8) / 50 (5.6)  0.88 (0.55 – 1.41) |  |
| 6q25 | rs9364554 | CC | 166 (58.7) / 214 (67.5)  1.00 (ref) | 384 (62.0) / 624 (70.4)  1.00 (ref) |  |
|  |  | CT | 98 (34.6) / 92 (29.0)  1.37 (0.97 – 1.95) | 217 (35.1) / 227 (25.6)  **1.55 (1.23 – 1.94)** | 0.09 |
|  |  | TT | 19 (6.7) / 11 (3.5)  **2.23 (1.03 – 4.81)** | 18 (2.9) / 35 (4.0)  0.83 (0.47 – 1.49) |  |
| **11q13** | **rs10896449** | GG | 142 (51.3) / 120 (38.7)  1.00 (ref) | 293 (47.3) / 390 (44.1)  1.00 (ref) |  |
|  |  | AG | 118 (42.6) / 146 (47.1)  **0.68 (0.48 – 0.96)** | 263 (42.5) / 398 (45.0)  0.88 (0.71 – 1.09) | **0.02** |
|  |  | AA | 17 (6.1) / 44 (14.1)  **0.33 (0.18 – 0.60)** | 63 (10.1) / 97 (11.0)  0.86 (0.61 – 1.23) |  |
| 11q13 | rs12793759 | GG | 149 (52.1) / 193 (61.3)  1.00 (ref) | 375 (59.4) / 568 (63.1)  1.00 (ref) | 0.45 |
|  |  | AG + AA | 137 (47.9) / 122 (38.7)  **1.45 (1.05 – 2.01)** | 256 (40.6) / 332 (36.9)  1.17 (0.95 – 1.44) |  |
| 17q21 | rs4430796 | CC | 77 (27.3) / 100 (31.8)  1.00 (ref) | 165 (26.4) / 283 (31.5)  1.00 (ref) |  |
|  |  | CT | 139 (49.3) / 168 (53.3)  1.07 (0.74 – 1.56) | 308 (49.2) / 439 (48.9)  1.20 (0.94 – 1.53) | 0.49 |
|  |  | TT | 66 (23.4) / 47 (14.9)  **1.82 (1.13 – 2.94)** | 153 (24.4) / 175 (19.5)  **1.50 (1.12 – 2.00)** |  |
| 17q24 | rs1859962 | GG | 96 (34.3) / 93 (29.5)  1.00 (ref) | 197 (31.8) / 258 (29.1)  1.00 (ref) |  |
|  |  | GT | 139 (49.6) / 144 (45.7)  0.94 (0.65 – 1.35) | 290 (46.9) / 431 (48.5)  0.88 (0.69 – 1.12) | 0.14 |
|  |  | TT | 45 (16.1) / 78 (24.8)  **0.56 (0.35 – 0.89)** | 132 (21.3) / 199 (22.4)  0.87 (0.65 – 1.16) |  |

*SNPs presented are those that are statistically significantly associated with prostate cancer among men aged ≤ 60 years
