# Supplementary material for: Characterization of SNPs Associated with Prostate Cancer in Men of Ashkenazic Descent from the Set of GWAS Identified SNPs: Impact of Cancer Family History and Cumulative SNP Risk Prediction
Source: PLoS One. 2013 Apr 3;8(4):e60083. doi: 10.1371/journal.pone.0060083 (PMC3616024; doi:10.1371/journal.pone.0060083)
Supplement: Table S4 — Associations of GWAS SNPs with Risk of Prostate Cancer stratified by Family History of Prostate Cancer and Other Cancers. (DOC) [file pone.0060083.s004.doc]

**Supplemental Table S4. Associations of GWAS SNPs with Risk of Prostate Cancer stratified by Family History of Prostate Cancer and Other Cancers**

1. First-degree Family History of Prostate Cancer

| Chrom | dbSNP | Genotype | No Family History of Prostate Cancer* | Positive Family History of Prostate Cancer* | P for  Interaction |
| --- | --- | --- | --- | --- | --- |
|  |  |  | Cases (%) / Controls (%)  OR (95% CI) | Cases (%) / Controls (%)  OR (95% CI) |  |
| 2p21 | rs1465618 | GG + AG | 538 (94.9) / 669 (97.6)  1.00 (ref) | 222 (96.9) / 160 (98.2)  **2.30 (1.83 – 2.91)** | 0.74 |
|  |  | AA | 29 (5.1) / 23 (2.4)  **2.38 (1.35 – 4.19)** | 7 (3.1) / 3 (1.8)  **4.24 (1.09 – 16.61)** |  |
| 6q25 | rs9364554 | CC | 398 (61.4) / 711 (69.2)  1.00 (ref) | 152 (59.8) / 127 (72.2)  **2.03 (1.55 – 2.66)** | 0.33 |
|  |  | CT + TT | 250 (38.6) / 316 (30.8)  **1.36 (1.11 – 1.68)** | 102 (40.2) / 49 (27.8)  **3.51 (2.43 – 5.06)** |  |
| 8q24 | rs6983267 | TT | 141 (21.4) / 280 (26.9)  1.00 (ref) | 56 (21.7) / 45 (25.7)  **2.48 (1.59 – 3.88)** | 0.14 |
|  |  | GT | 316 (47.9) / 525 (50.4)  1.16 (0.90 – 1.49) | 108 (41.9) / 96 (54.9)  **2.08 (1.47 – 2.94)** |  |
|  |  | GG | 203 (30.8) / 236 (22.7)  **1.71 (1.29 – 2.26)** | 94 (36.4) / 34 (19.4)  **5.11 (3.27 – 7.99)** |  |
| 17q24 | rs1859962 | GG | 195 (30.1) / 304 (29.5)  1.00 (ref) | 98 (39.0) / 47 (27.0)  **3.13 (2.11 – 4.66)** | 0.09 |
|  |  | GT | 316 (48.8) / 487 (47.3)  1.04 (0.82 – 1.31) | 113 (45.0) / 88 (50.6)  **1.96 (1.40 – 2.74)** |  |
|  |  | TT | 137 (21.1) / 238 (23.1)  0.91 (0.69 – 1.20) | 40 (15.9) / 39 (22.4)  1.59 (0.98 – 2.57) |  |
| **19q13** | **rs8102476** | CC | 267 (40.3) / 396 (38.0)  1.00 (ref) | 129 (50.0) / 63 (35.8)  **2.99 (2.12 – 4.22)** | **0.02** |
|  |  | CT + TT | 395 (59.7) / 646 (62.0)  0.92 (0.75 – 1.13) | 129 (50.0) / 113 (64.2)  **1.63 (1.20 – 2.20)** |  |
| 22q13 | rs5759167 | AA + AC | 476 (71.8) / 778 (75.0)  1.00 (ref) | 178 (69.8) / 129 (72.9)  **2.16 (1.68 – 2.80)** | 0.94 |
|  |  | CC | 187 (28.2) / 259 (25.0)  1.18 (0.94 – 1.48) | 77 (30.2) / 48 (27.1)  **2.52 (1.72 – 3.70)** |  |

*SNPs presented are those that are statistically significantly associated with prostate cancer at α=0.05 in one of the strata defined by family history

1. **Family History of Lung Cancer** (first or second-degree)

| Chrom | dbSNP | Genotype | No Family History of  Lung Cancer* | Positive Family History of  Lung Cancer* | P for  Interaction |
| --- | --- | --- | --- | --- | --- |
|  |  |  | Cases (%) / Controls (%)  OR (95% CI) | Cases (%) / Controls (%)  OR (95% CI) |  |
| **4q22** | **rs17021918** | CC | 342 (46.9) / 439 (44.8)  1.00 (ref) | 104 (55.0) / 82 (37.3)  **1.55 (1.12 – 2.14)** | **0.03** |
|  |  | CT | 301 (41.2) / 435 (44.3)  0.91 (0.74 – 1.12) | 66 (34.9) / 111 (50.5)  0.77 (0.55 – 1.08) |  |
|  |  | TT | 87 (11.9) / 107 (10.9)  1.05 (0.75 – 1.44) | 19 (10.1) / 27 (12.3)  0.87 (0.47 – 1.60) |  |
| 6q25 | rs9364554 | CC | 441 (61.3) / 689 (69.8)  1.00 (ref) | 109 (59.6) / 149 (69.0)  1.11 (0.84 – 1.47) | 0.94 |
|  |  | CT + TT | 278 (38.7) / 298 (30.2)  **1.42 (1.15 – 1.74)** | 74 (40.4) / 67 (31.0)  **1.61 (1.13 – 2.31)** |  |
| 8q21 | rs1512268 | GG | 213 (29.1) / 319 (31.9)  1.00 (ref) | 40 (21.2) / 69 (31.4)  0.83 (0.53 – 1.28) | 0.27 |
|  |  | AG | 382 (52.2) / 488 (48.9)  1.17 (0.94 – 1.47) | 111 (58.7) / 110 (50.0)  **1.44 (1.05 – 1.99)** |  |
|  |  | AA | 137 (18.7) / 192 (19.2)  1.02 (0.77 – 1.36) | 38 (20.1) / 41 (18.6)  1.35 (0.83 – 2.19) |  |
| 8q24 | rs6983267 | TT | 154 (21.1) / 277 (27.8)  1.00 (ref) | 43 (22.8) / 48 (22.0)  **1.66 (1.04 – 2.64)** | 0.15 |
|  |  | GT | 341 (46.7) / 501 (50.2)  1.20 (0.94 – 1.53) | 83 (44.2) / 120 (55.1)  1.14 (0.80 – 1.61) |  |
|  |  | GG | 235 (32.2) / 220 (22.0)  **1.90 (1.44 – 2.50)** | 62 (33.0) / 50 (22.9)  **2.14 (1.40 – 3.29)** |  |

*SNPs presented are those that are statistically significantly associated with prostate cancer at α=0.05 in one of the strata defined by family history

1. **Family History of Breast Cancer** (first or second-degree)

| Chrom | dbSNP | Genotype | No Family History of  Breast Cancer* | Positive Family History of  Breast Cancer* | P for  Interaction |
| --- | --- | --- | --- | --- | --- |
|  |  |  | Cases (%) / Controls (%)  OR (95% CI) | Cases (%) / Controls (%)  OR (95% CI) |  |
| 6q25 | rs9364554 | CC | 438 (62.7) / 646 (69.5)  1.00 (ref) | 112 (55.2) / 192 (70.3)  0.80 (0.61 – 1.04) | 0.06 |
|  |  | CT + TT | 261 (37.3) / 284 (30.5)  **1.30 (1.06 – 1.61)** | 91 (44.8) / 81 (29.7)  **1.55 (1.11 – 2.14)** |  |
| 10q11 | rs10993994 | CC | 157 (22.3) / 244 (26.3)  1.00 (ref) | 37 (18.1) / 67 (24.4)  0.81 (0.51 – 1.27) |  |
|  |  | CT | 351 (49.8) / 460 (49.5)  1.20 (0.93 – 1.53) | 99 (48.3) / 148 (53.8)  0.97 (0.70 – 1.34) | 0.18 |
|  |  | TT | 197 (27.9) / 225 (24.2)  **1.34 (1.01 – 1.78)** | 69 (33.7) / 60 (21.8)  **1.70 (1.13 – 2.55)** |  |
| **11q13** | **rs10896449** | GG | 323 (46.7) / 400 (43.4)  1.00 (ref) | 112 (54.9) / 110 (40.2)  1.21 (0.90 – 1.66) | **0.01** |
|  |  | AG + AA | 369 (53.3) / 522 (56.6)  0.87 (0.71 – 1.07) | 92 (45.1) / 164 (59.9)  **0.63 (0.46 – 0.84)** |  |
| 22q13 | rs5759167 | AA | 143 (20.1) / 249 (26.5)  1.00 (ref) | 43 (20.8) / 62 (22.6)  1.13 (0.73 – 1.77) |  |
|  |  | AC | 372 (52.3) / 459 (48.9)  **1.39 (1.08 – 1.79)** | 96 (46.4) / 137 (49.8)  1.12 (0.81 – 1.58) | 0.44 |
|  |  | CC | 196 (27.6) / 231 (24.6)  **1.47 (1.11 – 1.95)** | 68 (32.9) / 76 (27.6)  **1.46 (1.00 – 2.16)** |  |

*SNPs presented are those that are statistically significantly associated with prostate cancer at α=0.05 in one of the strata defined by family history
